# Annotations

**Published:** 1909-09-25

**Authors:** 


### ANNOTATIONS.

### Christian Science and Death.

Many readers will remember the so-called "Christian Science trial," which was held at the Old Bailey three summers ago, and came to an abortive end. The social position of the dead officer, and the fact that the prisoner was a qualified medical practitioner who had renounced his profession in favour of Christian Science, lent public interest to what was really a sordid and depressing exposure. From time to time the deaths are inquired into of credulous and misguided persons who have preferred the services of Christian Science "nurses" and "practitioners" to those of medical men. Lately a woman died at Worthing under such circumstances from phthisis, and the coroner's jury expressed their opinion of the matter in the following rider to their verdict: "that the practice of Christian Science, especially in cases of serious disease, is harmful, and greatly to be deprecated; and, further, that the taking of money for treatment is un-Christian." Legitimate medical practitioners are so often the victims of stupid and unnecessary riders from ignorant jurymen that some may feel glad to find a jury anxious to express in this form its views about Christian Science.

### An American Physician on the Liquor Problem.

In a recent issue of the New York *Medical Record* has appeared a short common-sense practical paper by Dr. Charles Rosenwasser on the Liquor Problem, which, in spite of the well-worn nature of the subject, deserves attention from English practitioners and sociologists. The author merely sets out to discuss the problem, and to make suggestions for its solution, in so far as it affects, and is affected by, present conditions in the State of New Jersey; but what he writes has a general application, which is not confined even to the United States. He maintains that the principal difficulties arising from the use of alcoholic beverages are due to two fundamental errors—each of which is also to be observed in this country. These are, firstly, impure products; and, secondly, an unsound system of handling these products. Whatever opposed views may be held upon the toxicity of alcoholic drinks when taken "in moderation," all medical authority is agreed that impure and improperly prepared alcoholic beverages are injurious. The State should therefore "so strengthen and enforce the pure food laws that those who drink will receive pure goods, and know just what they are drinking." As to the method of handling liquor, Dr. Rosenwasser sees in the American bar the greatest remediable evil; and his objections to the bar-system of dispensing strong drinks apply with equal force to the state of affairs in Britain. It is "especially vicious because it encourages men to drink when the stomach is empty, to drink hastily, and to drink more than they want." He would abolish the bar and the custom of "treating," and (by readjustment of liquor taxation) encourage the use of mild alcoholic beverages, such as beer and light wine, in place of ardent spirits. Meanwhile the campaign of education must continue with increased vigour, especially amongst the rising generation; and in this matter "we should lay greater stress upon the physical effects of alcohol than upon the moral effects, for we know there is nothing so conducive to healthy morals as a healthy body." With regard to the three theoretical solutions of the liquor problem—"Prohibition," "Local Option," and "High Licence"—all of which have been tried in various States of America and elsewhere, Dr. Rosenwasser can find nothing good to say of any one of them. "Prohibition encourages the use of whisky in place of wine and beer"; "Local Option means local prohibition and local strife"; "High licence is of no value, and may, indeed, work harm." The whole tone and temper of Dr. Rosenwasser's paper strike us as appropriate and practical, and his views and suggestions largely coincide with those which we have long held, and often expressed or implied.

### The London Medical Student.

Within the fast-dwindling ranks of "doctors of the old school"—whose portrait gallery has lately been enriched by a little sympathetic study from life by Henry de Vere Stacpoole in "The Doctor"—there may be some who can remember, even at this distance of time, the appearance of Albert Smith's "London Medical Student" in the days of their apprenticeship or "hospital walking." To them, perhaps, the boisterous fun of these wild caricatures of early Victorian hospital life and manners, will recall in sharp definition the far-away scenes of irresponsible studentship, which, in common with other recollections of old age, are often far more vivid and exact than the intervening memories of manhood and maturity. But to the curious-minded medical student of to-day, when in an idle moment at some bookstall, he turns up an old battered original, or buys a cheap miniature reprint, of this rather jaded work of humour, the coarse frolics of his predecessors must seem even more remote and unsubstantial than those of the immortal Ben Sawyer and Bob Allen. Indeed, Albert Smith's extravagant portraits—drawn from within by one who quitted Medicine for a jocular by-path of Literature—cannot compare with the no less farcical contemporary types of medical immaturity drawn by the clear, bold, spontaneous pencil of Dickens. Nevertheless, to those readers who have not only what is called an inquiring turn of mind, but also a moderately elastic sense of humour, we can recommend Albert Smith's little volume of exuberant sketches of medical student life as it was, more or less, in a past generation. It is perhaps at its best as a means of diversion for some of those long hours of waiting which are the portion of our profession, but especially of those amongst us whose midwifery practice is "extensive and peculiar."

